# Dr. Abercrombie on the Brain and Spinal Cord

**Published:** 1828-04-01

**Authors:** 


### Medico-Chirurgical Review,

### No. XVI.

[FASCICULUS III.]

FEBRUARY 9, 1828.

### Art. VII.

*Pathological and Practical Researches on Diseases of the Brain and the Spinal Cord.* By John Abercrombie, M.D. Fellow of the Royal College of Physicians of Edinburgh. Octavo, pp. 444. Edinburgh and London, Dec. 1827.

There are, or rather there ought to be, but two species of purely medical writings—the record of facts, and the deduction of principles from those facts. How is it, then, that medical science is so over-run with error? The reasons are but too obvious—more than half the facts are false—and nine-tenths of the deductions are illegitimate! We do not say that half our facts have been wilfully falsified; but, if the observation of a fact be incorrect, or the record of it distorted, it is the same for science whether the motive be good or bad which led to the error. Little did Cullen dream that, ere his bones were mouldered into dust, there would be a regularly organized school for the manufacture of false facts in medicine! As for illegitimate deductions from facts, (such as these facts are) they meet us at every step. It was easier, indeed, in all ages, to sit in the closet or library, spinning out theories, than to plod in the dissecting-room among the dead, or watch the phenomena of disease at the bedside of sickness. But the bad effects of theorising have now been so long conspicuous, that the medical profession is running into the opposite extreme, and, by shutting the door against all doctrine, has opened an immense and somewhat lucrative market for the sale of facts. It behoves the public to narrowly scrutinize these articles, now so plentiful and cheap; for assuredly there is no small proportion of counterfeit wares afloat.

Dr. Abercrombie has long distinguished himself by a careful observance of facts, the record of which has, from time to time, enriched our esteemed northern contemporary, through the medium of which journal, the major part of the volume before us was originally promulgated. That the papers published in the Ed. Journal have here assumed a much more systematic shape, and that the deductions from them are rendered far more useful, we are ready to grant—and, therefore, we think Dr. Abercrombie has done a service to the profession, and credit to himself, by this publication.

The volume is divided into four parts—the first three of which embrace diseases of the brain—the fourth, those of the spinal marrow. The cerebral affections are arranged under three classes—the INFLAMMATORY, the APOPLECTIC, and the ORGANIC. The inflammatory class will occupy our attention in the present article.

### INFLAMMATORY AFFECTIONS OF THE BRAIN.

This part of Dr. A.'s work is divided into seven sections, with an appendix. The author observes, with reason, that peculiar difficulties attend the investigation of this subject. The rapid effects which acute diseases of the sensorium produce on all the functions of this organ, render the patient unable to express his feelings correctly—and, hence, the proper symptoms of the disease are masked, or disguised, by that suspension of faculties, to which we give the name of oppression of the brain. This state has been naturally associated in the mind with PRESSURE on the organ—and enquiries have accordingly been directed towards the compressing cause. Effused fluid having, in many cases, been found, this fluid has been generally considered as explanatory of the symptoms, and there investigation has too often ended. This is exemplified in the case of acute hydrocephalus; but more accurate observation has taught us, that the condition which we denominate coma, with its usual symptoms, is not characteristic of any one state of brain, but may appear in states and diseases widely, if not diametrically, opposite in their nature. At all events, it is proved to have no necessary connection with an effused fluid, and that it may shew itself in simple inflammatory affections of the organ. Following the light thus obtained, the phenomena of cerebral inflammation are subjects of important investigation, especially as regards acute hydrocephalus, so called, since we are now pretty well authorised to conclude, that this disease is not a mere dropsical affection of the brain, but an inflammatory disease, terminating by effusion. This phlogosis varies in character, according as it is seated in the membranes, or the brain itself—and, also, according to its degree of activity, and the mode of its termination. Thus the dura mater, pia mater, or substance of the brain, may be the seats of inflammation—while the intensity may vary, from the most acute to the most chronic—its terminations being by serous effusion, deposition of false membrane, suppuration, or softening of the cerebral mass. The variety of phenomena resulting from these several seats and terminations, open a wide field for observation, both in the sick chamber and dissecting-room.

### SECT. I.—GENERAL VIEW OF THE SYMPTOMS INDICATIVE OF INFLAMMATION WITHIN THE HEAD.

Dr. A. acknowledges, in the outset, that he is incapable of distinguishing inflammation of the brain from that of its membranes—and this is the general opinion of the English Profession. The French pathologists, however, have lately endeavoured, and they say with success, to discriminate between the seats of phlogosis, even by the symptoms. Dr. A. considers the diagnosis as "not of much practical importance"—and hence his attention is directed to the phenomena which indicate the actual existence of inflammation, whether membranous or encephaloid.

1. The FIRST FORM—the phrenitis of systematic writers—is characterised by fever, insomnium, acute head-ache, intolerance of light, suffusion of eyes, maniacal delirium. Such a pure case, however, is rarely met with, except from the abuse of spirituous liquors, insolation, or as a supervention on fever or mania;—still more rarely after injuries of the head. It is probable that this form is the result of primary membranous inflammation. When fatal, the vital powers are rapidly exhausted by the high excitement, without much disorganization of the parts affected :—hence the dissections of such cases are unsatisfactory—if not deceptive. By the following passage, we should be induced to suppose Dr. A. is describing delirium tremens, though he does not say so.

"There is an affection of frequent occurrence, which perhaps may be referred to this head. It is characterised by a peculiar aberration of mind without any complaint of pain. There is a remarkable restlessness, quickness and impatience of manner, obstinate watchfulness, and incessant rapid talking, the patient rambling from one subject to another, with little connection, but often without any actual hallucination; he knows those about him, and generally answers distinctly questions that are put to him. There is a rapid pulse, but without the other symptoms of fever; and the disease is apt to be mistaken by a superficial observer for mania, and consequently to be considered as not being attended with danger. But it is an affection of very great danger, and is often very rapidly fatal. The nature of it is obscure, and the appearance on dissection is rather unsatisfactory; it consists chiefly of a highly vascular state of the Pia Mater, without any actual result of inflammation."

2. In the SECOND FORM, a sudden attack of convulsions, preceded or not by indisposition, is the first symptom that excites alarm. The convulsion is generally long and severe—in some cases followed by coma, proving fatal in a few days—in others, the paroxysm recurs frequently at short intervals, the patient being sensible in the intermediate periods, and complaining of headach, till, after twelve or twenty-four hours, coma supervenes. From this coma there is occasionally a complete recovery for several days, when, without any warning, the convulsion returns, and ends in fatal coma.

"In a very interesting modification of this form of the disease, the convulsion is confined to one side of the body, or to one limb, and is usually followed by paralysis of the part affected; and in some cases, the first symptom is a sudden attack of paralysis without the preceding convulsion. These cases are remarkable from their resemblance to the ordinary attack of hemiplegia. It will appear in the sequel, that they are often connected with inflammation of a small defined part of the cerebral substance; that the attack may be so sudden as precisely to resemble the paralytic attack from other causes; and that the disease in the brain may not have advanced beyond the state of simple inflammation, while the symptoms have gone through the usual course, and have terminated in fatal coma. In general, however, the disease in such cases will be found to have advanced to suppuration, or to the ramollissement or peculiar softening of the cerebral substance, to be afterwards more particularly referred to; while, on the other hand, in some very interesting cases of this class, the inflammation will be found to have been entirely seated in the membranes."

3. THIRD FORM. This is most commonly seen in children, though occasionally in adults. It is generally preceded by a day or two of languor and peevishness, and followed by fever. The patient is oppressed, and unwilling to be disturbed—complains of acute pain in some part of the head, with flushing of the face and impatience of light. In many, there is sickness of stomach—in some not. Sometimes the pain is in the neck—or even in the arms or other parts of the body. The pupil is usually contracted—the eye morbidly sensible, and sometimes suffused—tongue white but moist; sometimes clean—sleep disturbed—grinding of the teeth—bowels generally obstinate, though often natural. After some days, slight delirium appears, at first transient, or only at night—sometimes amounting to coma. In some cases, there appears a peculiar forgetfulness, the patient using one word for another, or misnaming persons or things, bearing no resemblance to delirium. These symptoms are followed by tendency to sleep, ending soon in coma. While this scene is passing, the pulse, at first frequent, usually falls to the natural standard or below it—the pain becomes less violent—eye loses its sensibility, becoming dull and vacant, often with squinting and double vision. The pulse from this depressed state, often rises in a few days or hours, to extreme frequency. Indeed, throughout the whole course of the disease it is very variable—so remarkable an inequality not being observable in other diseases. It is a symptom of serious import in all affections of the head. The patient is now in a state of perfect coma, sometimes with partial paralysis, and in a few days death closes the scene. The duration of the malady is very various—from five or six days to three weeks. At some period of its progress, there is generally a signal remission, giving sanguine but deceitful hopes of recovery. In very young children, who cannot describe their feelings, this form of the disease is characterized by fever, flushing, restlessness, screaming, and sometimes vomiting, succeeded, in a few days, by stupor and squinting, the pulse coming down as the stupor appears.

4. The FOURTH FORM of cerebral inflammation has been most frequently observed in young persons towards the age of puberty and upwards.

"It begins like a slight feverish disorder, and for a considerable time excites no alarm; there is slight headach, with general uneasiness of the limbs, impaired appetite, and disturbed sleep; the tongue is foul, and the pulse slightly frequent, probably from 96 to 100. After a few days the complaint appears to be going off; but, at our next visit, we are disappointed to find the patient complaining as much as at first. More active treatment is then adopted, and there is again an appearance of amendment; the tongue perhaps becomes clean, there is some appetite, and better sleep; but there is still some complaint of headach, which varies much in degree from one day to another, never severe, but never quite gone; the pulse continuing a little frequent. Amid these remissions and aggravations, eight or ten days may pass before the disease has assumed any decided character. It is not perhaps before the sixth or seventh day, that even an attentive observer begins to remark, that the degree of headach, though not severe, is greater and more permanent than corresponds with the general symptoms of fever; that the tongue is becoming clean, the pulse coming down, and the appetite improving, while the headach continues, with an unwillingness to be disturbed, and a degree of oppression which is not accounted for by the degree of fever. In this manner the disease may go on for several days more, until, perhaps about the 12th or 14th day, the pulse suddenly falls to the natural standard, or below it, while the headach is increased, with an evident tendency to stupor. This instantly marks a head affection of the most dangerous character, and the patient now lies for several days in a state of considerable stupor, sometimes with convulsion, often with squinting and double vision. The pulse then begins to rise again, and about this time there is frequently a deceitful interval of apparent amendment, sometimes the squinting goes off, and the eye appears quite natural, the stupor is lessened, and the patient appears easy and intelligent, but soon relapses into perfect coma, and dies in three or four days."

5. The FIFTH FORM of the disease is usually observed in adults, and begins with violent head-ache unaccompanied by fever. The patient lies in bed oppressed and unwilling to be disturbed, or tossing about from the violence of the pain. The pulse is natural, or even below 60—face sometimes flushed, sometimes pale—eye sometimes natural, in others morbidly sensible, with contracted pupil. The headache is usually acute and deep-seated, frequently appearing to shoot from temple to temple, or is referred to one ear. Sometimes vomiting occurs. Delirium frequently appears at an early period, varying in intensity from day to day, till, in five or six days, it passes into coma, the pulse continuing from 70 to 80 all the time. Sometimes the vision is unaffected—sometimes there is squinting with double vision. These latter symptoms occasionally disappear to return no more, but the disease goes on to a fatal termination. In every case, there is more or less delirium; but sometimes it is slight and transient. This condition, when unaccompanied by fever, is always characteristic of a dangerous affection of the brain. The speech is sometimes embarrassed, apparently from want of recollection of words. There is generally more or less of coma towards the close of the disease.

In all these forms, there is a great variety in the symptoms, and much attention is necessary, in order that the practitioner may not be thrown off his guard against the insidious nature of the disease. "Even in those cases which have assumed the most formidable aspect, every alarming symptom may subside," and during a deceitful interval, we indulge sanguine hopes—nay, the patient will sometimes dismiss his medical attendant. He falls into a drowsy state, which is considered favourable—he then sleeps almost constantly—and, in another day this sleep terminates in coma!

In this description, our author informs us that he has been entirely PRACTICAL. He has not stopped to inquire whether all these forms are to be considered as primary idiopathic affections of the brain—or whether some of them may be looked upon as secondary or symptomatic. There can be no doubt that many of them are secondary, supervening on other diseases, especially on continued fever—scarlatina—hooping-cough—measles—pneumonia—phthisis, and diseases of the kidney. The following is a brief summary of those symptoms which, in the course of any disease, indicate a tendency to dangerous cerebral affection.

"IN THE HEAD.—Violent headach with throbbing and giddiness—tinnitus—sense of weight and fullness—stupor—a great propensity to sleep. In many obscure and insidious cases, a constant feeling of giddiness is the only remarkable symptom.

"IN THE EYE.—Impatience of light—unusual contraction or dilatation of the pupil—double vision—squinting—blindness—distortion of the eyes outwards—paralysis of the muscles of the eyelids, producing, according to the muscle that is affected, either the shut eye, or the gaping eye—transient attacks of blindness or double vision—objects seen that do not exist—a long-sighted person suddenly recovering ordinary vision.

"IN THE EAR.—Transient attacks of deafness—great noise in the ears—unusual acuteness of hearing.

"IN THE SPEECH.—Indistinct or difficult articulation—unusual quickness of speech, or unusual slowness.

"IN THE PULSE.—Slowness and remarkable variations in frequency.

"IN THE MIND.—High delirium—transient fits of incoherence—peculiar confusion of thought, and forgetfulness on particular topics.

"IN THE MUSCLES.—Paralytic and convulsive affections—sometimes confined to one limb, or even part of a limb ; and a state of rigid contraction of particular limbs.

"IN THE URINE.—There frequently occurs a remarkable diminution of the secretion—sometimes nearly amounting to complete suppression; and connected with this diminution there is often a frequent desire to pass urine, occasioned probably by the increased acrimony, as the quantity diminishes."

So much for symptomatology—an important division of the study of diseases. We have presented a very full view of this part of our author's work, as it may prove a useful reference in the hurry of practice. We must now proceed to the second section, embracing the seats and terminations of the disease.

### SECT. II.—SEATS AND TERMINATIONS.

The varieties in the seat of the inflammation, may be referred to the following heads—dura mater—pia mater and arachnoid—substance of the hemispheres—dense white matter forming the central parts of the brain. In respect to the terminations, the disease may end fatally in the INFLAMMATORY STAGE, whether the inflammation be seated in the brain itself or in its coverings. In the most distinctly marked cases of such early termination, however, the phlogosis has been found in the cerebral substance. The other terminations are, as mentioned a few pages back, by SEROUS EFFUSION—DEPOSITION OF FALSE MEMBRANE—SUPPURATION—SOFTENING. On each of these terminations, the author makes a few remarks, and then proceeds to illustration by numerous cases.

1. *Serous Effusion.* Too much importance was attached to this by the earlier investigators, as if it alone constituted the disease called acute hydrocephalus. The symptoms were ascribed to the compressing influence of the effused fluid, and the practice was consequently directed to the removal of the effect, while the cause was neglected. It is now very generally admitted that the effusion in acute hydrocephalus is one of the terminations of inflammation, though there are certainly other causes from which serous effusion in these parts may arise.

Increased effusion from a serous membrane appears to take place under two very different conditions of the part. FIRST—from inflammation of the membrane itself or adjacent structures, as we see in the cavities of the pleura and peritoneum. The effused fluid varies very much, being limpid, opake, milky, flocculent, or nearly purulent. Who can tell on what these varieties depend? Dr. A. does not seem inclined to undertake this task—but he thinks we are authorised to conclude, that these various fluids, which are found in the head as well as in other cavities of the body, are the products of inflammation, in all those acute affections included under the term ACUTE HYDROCEPHALUS.

But there is another source of serous effusion entirely distinct from the above, viz. interruption of venous circulation. In this manner, we see a tightly bandaged limb become œdematous below the seat of pressure—and anasarca of the whole or part of a limb produced by the pressure of tumours—while ascites results from induration of the liver. "Such a state of impeded circulation evidently takes place in the brain, from a variety of causes; such as pressure of tumours, chronic disease of the sinuses, tumours on the neck, certain diseases of the lungs and of the heart—and, probably, from that very remarkable condition of the brain, to which I have proposed to give the name of simple apoplexy." From serous effusions produced by such causes as these, probably arise those affections which have been called CHRONIC HYDROCEPHALUS, and serous APOPLEXY.

2. *Deposition of False Membrane.* This is a product of inflammation as well ascertained as effusion. It is found sometimes between the dura mater and bone—sometimes between the dura mater and arachnoid—but most commonly it is under the arachnoid, where it is often found of great extent, communicating a yellow colour to a considerable surface of the hemisphere. It is occasionally found within the ventricles, covering the surface of the choroid plexus—and a very common seat of it is the upper surface of the tentorium.

3. *Suppuration.* A thin uniform layer of puriform matter is often found under the arachnoid, and occasionally betwixt the arachnoid and the dura mater, or even betwixt the dura mater and bone. It is also to be met with in distinct small cavities formed by partial adhesions of the membranes to the bone or to each other. It is occasionally found in the ventricles.

5. *Ramollissement.* We think Dr. A. might just as well have used the English term SOFTENING, as the French one *ramollissement*, which conveys precisely the same meaning. This morbid condition of the brain consists in a part of the cerebral substance being broken down into a soft pulpy mass, retaining its natural colour, but having lost its cohesion and consistence. It differs entirely from suppuration, having neither the colour nor the fetor of pus—for the white parts of the brain, in which it is most commonly observed, retain their pure milky whiteness. The corpus callosum, fornix, and septum lucidum, are the more usual seats of this peculiar disorganization.

In former communications, Dr. A. noticed this disease, and had no hesitation in attributing it to preceding inflammation. Since that time, the subject has undergone laborious investigation by the French pathologists, some of whom, especially M. Rostan, look upon this affection of the brain as one *sui generis*—sometimes, but not generally, the result of inflammation. Dr. A. now thinks that it occurs under two modifications, differing essentially from each other.

"In the cases of M. Rostan, the disorganization was observed chiefly in the external parts of the brain; it occurred almost entirely in very old people, few of his cases being under sixty years of age, many of them seventy, seventy-five, and eighty. It was found in connexion with attacks of a paralytic or apoplectic kind, many of them protracted; and was often found combined with extravasation of blood, or surrounding old apoplectic cysts. On the contrary, the affection which I had been anxious to investigate, was found chiefly in the dense central parts of the brain, the fornix, septum lucidum, and corpus callosum, or in the cerebral matter immediately surrounding the ventricles; and occurred in persons of various ages, but chiefly in young people and in children. It took place in connexion with attacks of an acute character, chiefly the character of acute hydrocephalus; and it was in many cases distinctly combined with appearances of an inflammatory kind, such as deep redness of the cerebral matter surrounding it, suppuration bordering upon it, and deposition of false membrane in the membranous parts most nearly connected with it. We may even observe in different parts of the same diseased mass, one part in the state of ramollissement, another forming an abscess, while a third retains the characters of active inflammation, and probably exhibits, as we trace it from one extremity to the other, the inflamed state passing gradually into the state of softening. Remarkable examples of this will be given in the sequel, and another of a different nature, in which an opening in the septum lucidum produced by the ramollissement, was entirely surrounded by a ring of inflammation. This is the affection which I have endeavoured to investigate, and which I consider as one of primary importance in the pathology of acute affections of the brain, and upon the grounds now shortly referred to, I cannot hesitate to consider it as a result of inflammation."

These facts being compared with the observations of M. Rostan, Dr. A. thinks we may arrive at a principle by which the apparent difference may be reconciled. Dr. A. considers the peculiar softening of cerebral matter as analogous to gangrene in other parts of the body—and that, like gangrene, it may arise from two very different causes—inflammation, and failure of the circulation from disease of the arteries. The former (phlogosis) Dr. A. conceives to be the origin of the affection which he has described—and the latter (defect of circulation) to be the source of the appearances described by M. Rostan. If this doctrine be admitted, the difficulty is removed. Gangrene from inflammation is familiar to every one—and equally familiar, though very different in origin and concomitant symptoms, is gangrene from a diseased state of the arteries. Ossification of the cerebral arteries is very common in elderly people —and, indeed, M. Rostan distinctly points to this, as the cause of his *ramollissement.* In young people, however, and after acute diseases, we may fairly attribute the phenomenon in question to inflammation. It is often combined with suppuration in other parts of the brain, and still oftener with effusion into the ventricles. In some cases it is, however, confined to a very small spot, as the septum lucidum and fornix, without any other disease in the encephalon.

6. *Chronic Form of Encephalitis.* Among the terminations of the chronic form, we may remark thickening of the membranes, contraction and obliteration of the sinuses, caries of the bones, and some other affections of the external parts. In respect to acute hydrocephalus, we cannot but admit, that the serous effusion is only one of the terminations of that inflammatory condition of the brain. Some of the other terminations are scarcely less frequent—especially the softening of the central parts, which is sometimes met with as the only morbid appearance, and is found combined with the effusion in a very large proportion of the ordinary cases of hydrocephalus. Other cases, in which the symptoms closely resemble those of hydrocephalus, will be found to terminate by the undefined suppuration, or by this combined with serous effusion, or with the ramollissement of the central parts. In fact, we but seldom meet with any one of the terminations uncombined—and it is impossible to anticipate, by the symptoms, in what way the inflammation may terminate in any given case.

Of these various terminations, Dr. Abercrombie has given numerous examples—these cases occupying the greater portion of the volume under review. It is not our intention to attempt the exhibition of any specimens of the cases. We shall entirely confine ourselves, in this analysis, to an enumeration of principles and precepts.

MENINGITIS. Passing over the illustrations of inflammation of the dura mater, one instance only of which, as purely idiopathic, has been seen by our author, we come to inflammation of the arachnoid and pia mater, from which we shall extract the following passage.

"Inflammation of the arachnoid, and of the pia mater, may be taken together. It is very difficult to distinguish them in practice, and as the affections are generally combined, it is probable that no important purpose can be answered by attempts to discriminate between their symptoms. The disease terminates most commonly by a deposition of false membrane betwixt the arachnoid and the pia mater. When this is found to spread uniformly over the surface of the convolutions, we may suppose that it has been produced from the arachnoid; when it dips considerably between them, it is probable that the pia mater has been affected; but, in point of fact, it is very often remarked in these cases, that the pia mater presents a most intense degree of vascularity, even when there is no deposition betwixt the convolutions, while there is seldom any remarkable vascularity observed in the arachnoid. On this ground it has sometimes been doubted whether the arachnoid be really the seat of inflammation.

"Some degree of this affection frequently accompanies other acute diseases of the brain, but we very often find it entirely uncombined, so that we are enabled to mark the symptoms more immediately connected with it. In these, however, there does not appear to be any uniformity. In some cases, it comes on with head-ach, vomiting, fever, and impatience of light; but I think the more common form in which the attack takes place, is by a sudden and long continued paroxysm of convulsion. This is in some cases preceded by headach and vomiting, but in other cases comes on without any warning. The convulsion is generally long and severe; in some cases, it passes immediately into coma, which afterwards alternates only with a repetition of the convulsion, until the case is fatal in a few days. In other cases, there is recovery from the first convulsion, and the patient appears to be doing well for some time, perhaps for several days, but afterwards falls into coma, either with or without a recurrence of the convulsion. In other cases again, the convulsion does not come on till an advanced period of the disease."

ENCEPHALITIS. As Dr. Abercrombie does not consider it possible to distinguish inflammation of the brain from inflammation of the membranes—and as he does not think it would be of any utility to make the distinction, if we could, why has he dedicated a section to each subject—why has he detailed the cases, as ascertained by dissection, under two distinct heads? This inconsistency is only to be reconciled by a statement made in the beginning of the work—namely, that the facts which he has laid before the public, are to be regarded as distinct from the deductions which he has drawn from them. Thus, he may say, I cannot make out encephalitis from meningitis, by the symptoms during life. But I have given you numerous histories of the one and the other disease, as verified by dissection—and you are at liberty to employ these histories or facts as you please, in drawing the distinction, if you are able, or if you think it of any advantage. On this principle, we think Dr. Abercrombie has acted properly, and we applaud him for his conduct.

He remarks, that the symptoms attendant on encephalitis vary considerably, according to the extent and particular seat of the phlogosis. Thus, in some cases, we find head-ache, followed by high delirium, and this by coma—in others, a sudden attack of convulsion.

"A frequent and very important form of the disease is characterised by head-ach, followed by convulsion of one or more limbs, the affected limbs afterwards becoming paralytic. Other cases again assume a close resemblance to the ordinary attack of hemiplegia, so as scarcely to be distinguished from it; and a very interesting feature of the affection in these cases is, that the disease in the brain may not have extended beyond the state of simple inflammation, though the symptoms have passed through their usual course, and have terminated in fatal coma.

"In the progress of the disease, considerable modifications occur, arising from the various ways in which the inflammation terminates. In this respect we are chiefly to attend to the following varieties.

"I. *It may be fatal in the inflammatory stage;*—a certain defined portion of the cerebral substance, presenting the appearance of deep redness, without any change of structure.

"II. *The simple ramollissement;* which consists in a part of the brain being broken down into a soft pulpy mass, retaining the natural colour of the part, without any appearance of suppuration, and without fœtor. This condition we often find as the only morbid appearance, but we frequently find it combined with the former, one portion of the diseased mass presenting the deep red colour, while another is in the state of ramollissement.

"III. The preceding appearance mixed with a proportion of purulent matter.

"IV. *The undefined suppuration.* This might perhaps be considered as a modification of the former, but with the purulent matter predominating in quantity. It presents a large ragged undefined cavity, filled partly with fetid purulent matter, and partly with broken down cerebral substance, the surrounding substance being soft and disorganized.

"V. *The defined or encysted abscess.* This consists of a well defined regular cavity, filled with purulent matter, generally lined by a soft cyst, and surrounded by cerebral matter in a healthy state.

"VI. *Ulceration of the surface of the brain.*"

The sixth section, from page 124 to page 146, includes a great number of cases of ramollissement of the central parts of the brain, and hydrocephalus. In reviewing the facts illustrative of this last subject, Dr. A. makes some practical observations which are deserving of notice. He remarks, that all medical practitioners must have found instances of serous effusion in the head after death, where the symptoms did not lead to the suspicion of such an event.

"It is therefore not the mere presence of a certain quantity of fluid in the brain, that gives rise to the symptoms of hydrocephalus; and, on the other hand, we have seen a disease go through all the usual symptoms of hydrocephalus, and terminate fatally without any effusion. The fair conclusion from these facts appears to be, that the prominent symptoms in these cases are not the result of the effusion, but of that disease of the brain of which the effusion is one of the terminations. From a variety of facts which have been adduced, there seems little reason to doubt that this disease is of an inflammatory nature. If these conclusions shall be considered as well founded, it will follow, that our practice ought to be directed principally to subduing the inflammation at its earliest period, and preventing it from passing into effusion, and particularly from passing into ramollissement, which we have seen to be a fatal termination of the disease, even though of small extent and without any effusion."

In regard to the mere effusion itself, were the parts in an otherwise healthy state, there appears no reason to consider it as a hopeless affection. In other words, Dr. A. means to submit, that we have no good reason for doubting the possibility of absorption of serous fluid from the ventricles of the brain. We are warranted in this supposition, he thinks, both by the analogy of other serous cavities, and by what we actually see take place in the brain itself. In this last organ, we have proof that coagula of blood are gradually absorbed, both from the ventricles, and from cavities in the cerebral substance. Upon the whole, Dr. A. believes, that we have sufficient ground for receiving the following conclusions, in regard to this class of affections.

"1st, That in the ordinary cases of hydrocephalus, the coma and other symptoms attending it are not to be considered as the direct effect of the effusion, but of that morbid condition of the brain of which the effusion is the consequence.

"2d, That we have no certain mark which we can rely upon as indicating the presence of effusion in the brain. Slowness of the pulse followed by frequency, squinting, double vision, dilated pupil, paralytic symptoms, and perfect coma, we have seen exist without any effusion.

"3d, That all these symptoms may exist in connexion with a state of the brain, which is active, or simply inflammatory, while the disease is the subject of active treatment, and while by such treatment, adopted with decision at an early period, we have the prospect of arresting its progress in a considerable proportion of cases. The ground of prognosis in particular cases depends perhaps in a great measure upon the activity of the symptoms. The more they approach to the character of active inflammation, our prospect of cutting them short will be the greater; and the more they partake of the low scrofulous inflammation, it will be the less. In all of them, the period for active practice is short, the irremediable mischief being probably done at an early period of the disease."

These principles bear on the question—can hydrocephalus be cured? Whether the fluid can be absorbed, or the disease cured, after the effusion has taken place, must remain a matter of conjecture, since we have no certain diagnostic marks by which we can tell when fluid has actually been thrown out: but Dr. A. thinks we have every reason to believe that, in the ordinary cases of hydrocephalus, the absorption of the fluid, if it did take place, would in no respect improve the situation of the patient—because there would still remain that deep-seated disease of the central parts of the brain, which accompanies the effusion in a large proportion of cases, and which proves fatal without any effusion at all, yet with the ordinary symptoms of hydrocephalus.

### Etiology of Encephalitis and Meningitis.

The causes of these inflammations generally elude our observation. We see them often arise in the course of other febrile diseases, especially scarlatina—we see them follow injuries; (and this is probably a more frequent cause than is suspected, particularly in children)—suppressed evacuations, as of the menses, in young women of unsound constitutions—ischuria renalis, &c. The brain occasionally takes on this disordered condition in the advanced stages of phthisis—and, indeed, of any other chronic disease, especially in scrofulous constitutions. Hepatic disease—worms—and various affections of the bowels, are regarded by many as causes of encephalitis. Insolation is a very frequent cause, sometimes inducing an apoplectic state, which proves fatal in a few hours—more generally an inflammatory affection, assuming the character of mania—rarely paralysis. This cause is, of course, most operative in the warmer regions of the earth.

### Treatment.

The remedies, in this important class of diseases, are few and simple. The great object is to know *when* we have inflammation to treat, for, that ascertained, the indications are plain enough. But we are convinced—the more so, indeed, the longer we live—that, of all the causes of want of success in medical practice, the ignorance of pathology and diagnosis is the greatest. It is on this account that we dwell so much on these important points, because we know that the young practitioner is always eager to jump at once to the treatment, before he has clearly ascertained the nature of the disease on which he is so ready to open the magazine of therapeutics!

Our diagnosis formed, every thing depends on applying our remedies early and boldly. These remedies are, as every one knows—blood-letting, local and general, active purgation, cold to the head. The effects of blistering, in the early stage, are rather ambiguous. "When it is employed, it should always be on the back part of the head and neck," because then it does not interfere with the application of cold to the vertex. After the first activity of the disease is subdued, "blisters applied, in succession, to various parts of the head, and the upper part of the spine, appear to be, in many cases, extremely useful."

"Mercury has been strongly recommended in that class of cases which terminate by hydrocephalus, but its reputation seems to stand upon very doubtful grounds. In many cases, especially during the first or more active stage, the indiscriminate employment of mercury must be injurious. In the adaptation of the particular remedies to individual cases, we must of course be regulated by the age and habit of the patient, and particularly by the character of the disease in regard to activity. In those cases which assume the more acute or active forms, general bloodletting must be used in the most decided manner; while in the cases which assume a more chronic character, as many of the common cases of hydrocephalus, it has less control over the disease, and is not borne to the same extent. In all the forms of the disease, active purging appears to be the remedy from which we find the most satisfactory results; and although bloodletting is never to be neglected in the earlier stages of the disease, my own experience is, that more recoveries from head affections of the most alarming aspect take place under the use of very strong purging, than under any other mode of treatment. In most of these cases indeed full and repeated bleeding had been previously employed, but without any apparent effect in arresting the symptoms. The most convenient medicine for this purpose is the croton oil. In regard to local remedies, by far the most powerful is the application of cold. It may be applied in a continued manner by means of a bladder containing pounded ice mixed with a small quantity of water; but a still more effectual mode of applying it in the more acute cases, is by a stream of cold water directed against the crown of the head and continued for a considerable time, until the full effect be produced from it. Applied in this manner, it is a remedy of such power, that it requires to be used with much discretion. Under the operation of it, I have seen a very strong man thrown, in a very few minutes, into a state approaching to asphyxia, who immediately before had been in the highest state of maniacal excitement, with morbid increase of strength, defeating every attempt of four or five men to restrain him."

We shall here notice a most remarkable defect in Dr. Abercombie's work. The class of diseases in question comprehends hydrocephalus, on which subject Dr. Abercrombie dwells particularly, and recurs to again and again. Yet, in all his descriptions, he never once alludes to the singularly deranged state of the bowels—the highly depraved condition of the alvine excretions and secretions in that disease. The peculiar appearance of the stools in hydrocephalus is so well known, that the merest tyro recognizes them instantly—in fact, every practical man knows, that they are even denominated "hydrocephalic stools." How so important a feature of the disease could have escaped Dr. Abercrombie, who takes care to distinctly tell us, that he is "entirely practical" in his descriptions and observations, we cannot imagine! Nay, even in the catalogue of organs and parts, whose functions are deranged in this class of complaints, (page 15-16) Dr. Abercrombie has actually omitted entirely the whole and every of the digestive organs, while he has allotted an unusual space to the state of the urine! This omission appears to us so totally inexplicable, that we shall not attempt to account for it. It is possible that, if we were to wade through the individual cases, we might find the fact of this intestinal and hepatic derangement sometimes recorded; but this can be no excuse for omitting, in his general descriptions, one of the most important features of the disease. We confess that a reflection on this singular defect in Dr. Abercrombie's work, has somewhat shaken our confidence in his therapeutics. The man who could overlook derangement of the digestive organs in hydrocephalus, may very readily overlook some important remedies adapted to the correction of this derangement. The slur which is thrown upon mercury in hydrocephalus, and the high niche in which croton oil is placed, as a purgative, stagger us a little, as to the fact of our worthy Doctor being so "entirely practical" as he wishes us to believe. We suspect that he has a crotchet in his brain, as well as Clutterbuck, Abernethy, and some others whom we could name. These crotchets are always throwing people out of tune, and doing much mischief.

Under the treatment above described, Dr. A. assures us that he has seen many cases recover, which exhibited all the usual symptoms of the most dangerous affections of the brain—"and even the most advanced stages of them." These favourable cases, however, form but a very small proportion of the total number; although they hold out encouragement to perseverance, longer than is generally practised.

Thus ends our author's therapeutics, which are few and simple indeed. Without being advocates for polypharmacy, we may venture to remark that, although bleeding, purging, and cold to the head, are good remedies, they are not the only good ones. Knowing the efficacy of mercury in other inflammations, and having had repeated opportunities of witnessing its good effects in hydrocephalic inflammations, we cannot but directly question opinions respecting mercury. In this class of diseases, we give it not merely as a purgative, but with the view of affecting the constitution—that is, of setting all the glandular system into action. The biliary secretion, as we before observed, is highly depraved—and whether this be effect or cause of the cerebral irritation, it is of the greatest consequence to correct it. No medicine, we affirm, is so capable of doing this as mercury, given in appropriate doses every four or six hours. The most drastic purgation will not rectify this biliary derangement. It may carry off disordered secretions, but will not prevent their formation immediately afterwards. Neither do we think our author justified in passing, unnoticed, the powerful effects of antimony, digitalis, and pediluvia with mustard. Surely, Dr. Abercrombie must have seen the antiphlogistic effects of these remedies—and we speak from experience, also, when we maintain that, in such dangerous diseases, we should employ several means, all tending to the same end. All constitutions are not alike. In some, bleeding does not succeed so well as other means of arresting inflammatory action. And in all cases the auxiliaries above-mentioned, after the sanguineous depletion, accelerate the cure, and save the effusion of blood. The semicupium is a very useful measure, by drawing a great volume of blood to the vessels of the lower half of the body, and increasing the biliary secretion, which is generally defective as well as depraved.

With these few strictures on Dr. Abercrombie's work, we shall take our leave of him for the present. The extended analysis which we have given of the first part of his publication, occupying nearly half the volume, shews that we hold this talented author in high estimation. When the subject of apoplexy occupies our pages, we shall return to the work, and pay due respect to the second part of his Essay.

